# A Case for Cognitive Entrenchment: To Achieve Optimal Best, Taking Into Account the Importance of Perceived Optimal Efficiency and Cognitive Load Imposition

**DOI:** 10.3389/fpsyg.2021.662898

**Published:** 2021-07-27

**Authors:** Huy P. Phan, Bing Hiong Ngu

**Affiliations:** School of Education, University of New England, Armidale, NSW, Australia

**Keywords:** cognitive entrenchment, perceived zone of comfort, cognitive load theory, optimization, optimal best, optimal efficiency, Jose Mourinho

## Abstract

One interesting observation that we may all concur with is that many experts, or those who are extremely knowledgeable and well-versed in their respective domains of functioning, become “mediocre” and lose their “touch of invincibility” over time. For example, in the world of professional football, it has been argued that an elite football coach would lose his/her air of invincibility and demise after 10–15 years at the top. Why is this the case? There are different reasons and contrasting viewpoints that have been offered to account for this observed demise. One notable concept, recently introduced to explain this decline, is known as *cognitive entrenchment*, which is concerned with a high level of stability in one's domain schemas (Dane, 2010). This entrenchment or “situated fixation,” from our proposition, may act to deter the flexibility and/or willingness of a person to adapt to a new context or situation. Some writers, on this basis, have argued that cognitive entrenchment would help explain the demise of some experts and/or why some students have difficulties adapting to new situations. An initial inspection would seem to indicate that cognitive entrenchment is detrimental, potentially imparting evidence of inflexibility, difficulty, and/or the unwillingness of a person to adapt to new contexts (Dane, 2010). This premise importantly connotes that expertise may constrain a person from being flexible, innovative, and/or creative to ongoing changes. In this analysis, an expert may experience a cognitive state of entrenchment, facilitated in this case by his/her own experience, knowledge, and/or theoretical understanding of a subject matter. Having said this, however, it is also a plausibility that cognitive entrenchment in itself espouses some form of positivity, giving rise to improvement and/or achievement of different types of adaptive outcomes. Drawing from our existing research development, we propose in this conceptual analysis article that personal “entrenchment” to a particular context (e.g., the situated fixation of a football coach to a particular training methodology) may closely relate to three major elements: *self-cognizance of cognitive load imposition*, a *need for efficiency*, and the *quest for stability and comfort*. As we explore later, there is credence to accept the “positivity” of cognitive entrenchment—that by nature, for example, a person would purposively choose the *status quo* in order to minimize cognitive load imposition, optimize efficiency, and/or to achieve minimum disruption and a high level of comfort, which could then “optimize” his/her learning experiences. We strongly believe that our propositions, which consider eight in this article, are of significance and may, importantly, provide grounding for further research development into the validity of cognitive entrenchment.

## Introduction

Excessive practicing of a specified task may develop *expertise* and in-depth understanding of a subject matter. Consider, for example, a football player who practices the art of free kicks daily. Over time, his/her automated behavior of shooting for a goal would transform into an important entity of expertise. In the context of academic learning likewise, as one of the authors of this manuscript recalled, it was a matter of excessive practicing of mathematics questions (e.g., spending 3 h after school on a Thursday to solve simultaneous linear equations with two unknowns, *x* and *y*), say—do as many questions as possible so that this act, in itself, would result in automaticity. From this personal account, the premise is that ongoing practice (e.g., repeated solving of Algebra problems) would give rise to success and, importantly, the development of expertise. Limited practice, in contrast, would indicate the novice state of knowledge and understanding of a person.

Repeated practice, in-depth reading of unit materials, active participation and self-discovery, and seeking academic support from others may facilitate and enhance expertise development. Importantly subject expertise may account for and/or produce a number of different types of adaptive outcomes (Gobet, 2006; Nokes et al., 2010; Ericsson et al., 2018; Lane and Chang, 2018). For example, within the context of academic learning, the expert knowledge of a student is likely to play a pivotal role in helping to facilitate his/her comprehension and deep, meaningful understanding of a subject matter. By the same token, when compared with a novice learner, an expert can make sound, logical decisions and transfer appropriate solutions from one context to another similar context. A novice, in contrast, is more likely to experience a number of difficulties (e.g., his/her limited pedagogical knowledge, which could assist with problem-solving), which would limit his/her ability to flourish and perform academically and non-academically. A number of “deficits” may espouse a novice learner, such as his/her inability to find an appropriate solution for usage, and/or his/her perceived difficulty comprehending and making logical sense of a subject matter.

It is interesting to note that in a recent publication, Dane (2010) introduced a concept known as “cognitive entrenchment.” Cognitive entrenchment, in brief, refers to the expertise of a person in a specific domain of functioning and its subsequent impact on his/her learning processes, motivational beliefs, and/or performance outcomes (Dane, 2011; Schmid, 2017; Engelberg, 2018). Cognitive entrenchment, in this case, may arise from continuing practice of a person, sustained training, in-depth reading, rote learning, self-discovery, etc. An analysis of the proposition of Dane (2010) suggests that, indeed, cognitive entrenchment could pose serious problems, limiting a person from being innovative, creative, and/or flexible. However, the premise of our conceptual analysis counters the proposition of Dane (2010) of cognitive entrenchment and contends that expertise may have a number of advantages. One notable aspect, which forms the basis of this article is that rather than being negative, situated fixation to expert schemas may assist in the achievement of “perceived optimal efficiency” (Phan and Ngu, 2021c). Perceived optimal efficiency, in brief, is concerned with the perceived judgment, assessment, and decision making of a person of his/her expenditure of time and/or effort, as well as his/her utilization of resources in terms of efficiency or inefficiency. Five hours of tutorial support to assist a student to attain a basic understanding of linear equations with one unknown, x, would be considered inefficient. Usage of deep cognitive strategies (Graham and Harris, 1989; Heikkilä and Lonka, 2006; Senko and Miles, 2008) to gain in-depth understanding of a subject matter, in contrast, would be more efficient for the time and/or effort of a student.

In summary, from the mentioned theoretical account, cognitive entrenchment may have a number of contrasting implications for consideration. In this sense, aside from achieving a state of optimal efficiency (Phan and Ngu, 2021c), which is positive, we contend that situated fixation of expert schemas could potentially help reduce the imposition of the cognitive processing of information of a person (Sweller et al., 2011; Sweller, 2012). From existing literature (Sweller et al., 2011; Sweller, 2012), cognitive load imposition is a negative element that may cause ineffective learning and other achievement-related difficulties. In this analysis, the utilization of expert schemas (e.g., the cognitive entrenchment of expert schemas) would play a pivotal role in helping to reduce cognitive load imposition (e.g., extraneous cognitive load). Within the context of academic learning, having expert pedagogical knowledge would limit the reliance of a student on cognitive resources to comprehend, interpret, and understand unit materials. For example, from cognitive load theory, an instructional design that highlights the integration of multiple sources of information to eliminate the split-attention effect would, in this case, assist a learner by generating expert schemas across different domains (Sweller et al., 1990; Lee and Kalyuga, 2011). Thus, drawing from this brief introductory explanation, we argue the following: that *there is credence to consider the applicability and relevance of cognitive entrenchment* in educational context (Dane, 2010).

## Cognitive Entrenchment: A Brief Overview

*Cognitive entrenchment*, according to Dane (2010), is defined as “a high level of stability in one's domain schemas” (p. 583). Moreover, according to the definition of Dane (2010), “the schema stability characterizing cognitive entrenchment may emerge, at least in part, from the frequency with which experts tend to draw on their domain schemas” (p. 583). This description is interesting and suggests that there is an intimate association between cognitive entrenchment and the *expertise* of a person, or expert schemas, which in this case is defined as a high level of domain-specific knowledge and understanding, *via* means of personal experience (Benner, 1984; Dreyfus and Dreyfus, 1986; Charness and Schultetus, 1999; Ericsson, 2006; Anders Ericsson et al., 2007). The writing of Piaget (1963, 1990) has likewise referred to the term “schema” and its constructive formation *via* the psychological process of adaptation. One major distinction between experts and novices is related to the *nature of schema*—that is, expert schemas are much larger and more complex than novice schemas (Fiske and Taylor, 1991; Rousseau, 2001). In other words, the *theory of personal constructivism* of Piaget (1963, 1990) attests that expert learners have more complex and interrelated schemas than novice learners. In terms of academic learning, say, a 1st-year university student who has expert knowledge in Psychology would demonstrate his/her in-depth understanding of different topical themes. A student who has novice knowledge, in contrast, would achieve mediocre academic results and, likewise, exhibit a limited understanding of different topical themes.

The study of the nature of expertise has been extensive, detailing the intricacy and implications of expert schemas in different domains of functioning (e.g., the study of chess playing). One notable line of inquiry in this analysis relates to the effect of *cognitive load imposition* (Sweller et al., 2011; Sweller, 2012), which we explore later in the article. However, what is poignant is that there is clear and consistent evidence to affirm the benefits of having expertise or expert knowledge. Numerous daily life-related reasons may support and/or account for this testament. For example, complex domain schemas help expert learners make effective decisions, exhibit superior recall of information, perform well (e.g., academic performance in a test), and being able to engage in problem solving, which could successfully transfer to new contexts (Chi et al., 1988; Hoffman, 1992; Ericsson et al., 2018). In terms of academic learning of Chemistry, say, a student who has expert knowledge of molarity may skip a particular procedural step and, instead, demonstrate his/her ability to generate a two-step solution (Ngu and Yeung, 2012). Thus, unlike novice learners, experts can fast track their “use” of procedural steps involved, reflecting their practice of automaticity of comprehension, understanding, pattern recognition, etc. In this analysis, in terms of automaticity, an expert learner may recognize a specific pattern and, using his/her existing knowledge, skip one or more intermediate steps so that the solution becomes simpler (Blessing and Anderson, 1996). The underlying premise from this then is that the large repertoire of knowledge and skills of a subject content of a student, and/or his/her deep procedural knowledge and in-depth understanding would allow him/her to respond automatically to a problem posed—that is, in other words, to “practice” the act of automaticity. Students who are novices, in contrast, would find it somewhat difficult to comprehend a problem, and/or to identify an appropriate and relevant learning strategy or strategies for effective usage.

Having expert schemas as opposed to novice schemas is advantageous. One major distinction, in this analysis, entails the fact that expert learners would outperform novice learners in different domains of functioning (e.g., playing chess) for various reasons, which we previously mentioned and discussed (e.g., the ability to transfer understanding of a solution to a new context; Margulies, 1991; Puddephatt, 2003; Scholz et al., 2008). Despite this testament, interestingly, some scholars have argued that expertise in itself could serve as a double-edged sword, limiting a person from progressing forward despite the mentioned advantages and benefits (e.g., providing domain-specific knowledge to assist in problem solving). As Dane (2010, 2011) has attested, one possible caveat of expertise relates to the notion of cognitive entrenchment. According to Dane (2010, 2011) and some other scholars (e.g., Lewandowsky and Kirsner, 2000; Chi, 2006; Lewandowsky et al., 2007), acquired expert knowledge would play a pivotal role in limiting the *flexibility* of a person to certain aspects within his/her domain. For example, as Dane (2010, p. 583) highlights in his analysis, expert learners may often struggle to understand how novices approach identical or similar problems (Camerer et al., 1989; Hinds, 1999; Thaler, 2000; Birch and Bloom, 2007). Expert learners may exhibit a restricted ability and/or unwillingness to accommodate new rules and principles (Frensch and Sternberg, 1989; Marchant et al., 1991).

The theoretical account of Dane (2010, 2011) then, from our brief examination, contends the possibility that possessing expertise could closely associate with a number of deficits—for instance, an experience of an expert of difficulty and/or unwillingness to adapt to new contexts or situations, which then would negate his/her ability, insight, and motivation to be inventive, innovative, and creative. Thus, at first sight, the writing of Dane (2010, 2011) seems to support the potential negativity of expert schemas. The situated fixation of a person to a particular context may serve to weaken and/or negate his/her willingness to engage in innovation, creativity, advancement, etc. Perceived negativity of cognitive entrenchment is somewhat contentious, we contend, as this testament contradicts and differs from existing research development, which considers the potential positivity of expert schemas (Chi et al., 1988; Hoffman, 1992; Ericsson et al., 2018). Let us consider the premise of cognitive entrenchment and elucidate its nature with reference to professional football. Recently, Grech (2020) wrote an interesting article titled “Cognitive Entrenchment and the curious case of José Mourinho” (Source: https://footyanalyst.com/cognitive-entrenchment-and-the-curious-case-of-jose-mourinho/). Those who follow European football would know the name José Mourinho, who is widely considered to be one of the greatest and decorated managers of all time. José Mourinho has won more than 25 titles (e.g., two UEFA Champions League titles with two different teams from two different countries) but yet, more recently, he has been relatively modest in his successes. Some journalists and pundits have argued that the game has “moved on” and that José Mourinho is now a yesterday's man (Source: https://www.the42.ie/is-jose-mourinho-now-yesterdays-man-5140367-Jul2020/). In a webpage article published this year, journalist Tannoury (2020) wrote the following: “On the pitch, the tactics employed by Mourinho—irritatingly defensive set-ups and opportunistic play in attack, with long passes launched for the wingers or the lone striker—have been left behind by rivals such as Jurgen Klopp at Liverpool and Manchester City's Pep Guardiola. Now a younger generation of football managers, including RB Leipzig's 33-year-old coach Julian Nagelsmann, introduces new concepts that are evolving the game. Mourinho, so far, has not adapted” (Source: https://www.thenational.ae/sport/football/twenty-years-as-a-manager-for-jose-mourinho-this-season-could-be-his-most-important-yet-1.1086662).

The webpage article by Tannoury (2020) regarding the demise of José Mourinho is interesting, as it incorporates and details an important element that we previously described: the ability (vs. inability) and/or willingness (vs unwillingness) for a person to adapt to a new context and be inventive and creative. The assertion here is that the training methodology of José Mourinho is somewhat outdated and does not align with the modern game. The assessment of Grech (2020) of José Mourinho likewise is insightful, suggesting that the revered coach is experiencing a state of cognitive entrenchment. It is reasoned that “entrenchment” of José Mourinho with his expert knowledge in training methodology (i.e., the methodology of what is known as “tactical periodization”) and past successes have prevented him from making creative and innovative changes to new challenges. We postulate, too, that José Mourinho's self-confidence and personal conviction of his expertise may, indeed, account for his unwillingness and/or inflexibility to consider the possibility that other more “superior” training methodologies (e.g., the setting up of an attacking formation) could yield better success. This testament is interesting as it posits the tenet that situated fixation of knowledge and understanding may associate with and/or produce detrimental consequences.

Cognitive entrenchment (Dane, 2011; Schmid, 2017; Engelberg, 2018) may also explain students' negative learning experiences. Different academic learning contexts may support the argument of Dane (2010), such as the inability, inflexibility, and/or unwillingness of a student to adapt to the contextual environment. For example, let us consider a few in-class scenarios:

- A secondary school student (e.g., Student A) ignores the advice of his teacher and instead chooses to use a particular cognitive strategy that he is well-versed with (e.g., using a strategy that involves “drilling and practicing”). The insistence of a student to use a preferred cognitive strategy may be guided, in this case, by his personal experience, in-depth understanding, and previous successes.
- A university student (e.g., Student B) who has a strong foundation and expert knowledge of History insists that she would like to continue with this major, despite the objection of her family that this specialization would not yield many employment opportunities, career-related prospects, etc., in the future. The insistence of a student to specialize in History may arise from her previous academic accomplishments, personal interest, perceived value, etc.
- The preference of an elementary school student (e.g., Student C) to choose the Solar System as a topic for individual, in-class presentation. The purposive choosing of a student may, in this case, relate to his liking for Astronomy, consequently shaped by successful test results.

The three mentioned examples, in brief, highlight the potential negativity of expert schemas, which reflect the following: (i) unwillingness to consider adapting to a new learning situation (e.g., Student A), (ii) unwillingness and non-receptive of other viewpoints (e.g., Student B), and (iii) unwillingness and/or uncertainty to seek new knowledge (e.g., Student C). A common theme resonating across the three examples relates, in this case, to the “fixation” and preference of a student on the *status quo* and his/her unwillingness to vary and to consider an alternative. Our examples reflect the “negative” nature of cognitive entrenchment (Dane, 2011; Schmid, 2017; Engelberg, 2018), which emphasizes the impact of the expert knowledge in terms of content and/or procedural of a student.

### An Alternative Consideration: An Introduction Into the “Positivity” of Cognitive Entrenchment

The preceding sections have provided an overview of expert schemas and the importance of cognitive entrenchment (Dane, 2010, 2011). Situated fixation may compel a person to think, act, and behave in a manner that would be consistent with their personal experiences, knowledge, understanding, etc. Cognitive entrenchment, as detailed, may result in a number of maladaptive outcomes, such as the unwillingness and/or perceived difficulty of a person to change and adapt to a new context or situation. The case of José Mourinho, as some would argue, is a typical example of the potential negativity of cognitive entrenchment. Having said this, however, we do have an interesting and important question to ask, which forms the premise of our conceptual analysis: why is this the case, though—that is, why would a person feel inclined to remain with the *status quo*?

There are contrasting underlying reasons that may account for the “entrenchment” of a person to a particular context or course of action (e.g., the insistence of a student to major in History). In this analysis, at this stage, let us consider a few possible “positive” reasons as to why the act of cognitive entrenchment is warranted:

i. Personal experience of success instills self-confidence, satisfaction, and contentment, compelling a student to remain on course without any deviation. In the area of *student motivation* (Pintrich and Schunk, 2002; Schunk et al., 2008), for example, there is clear and consistent evidence to indicate that personal successes, reflecting both mastery and performance-based outcomes, form an important source of information, which then would heighten the sense of self-efficacy of a student for academic learning (Usher and Pajares, 2006; Pajares et al., 2007; Liem et al., 2008; Phan, 2012). Personal experience of continuing failures in a subject matter, in contrast, would weaken the sense of self-efficacy of a student, resulting in his/her underachievements and negative learning experiences.
ii. Considering the importance of *theory of personal cognition* of Piaget (1955, 1963), it is a natural tendency and/or likelihood that a student would seek to reach a mental state of equilibrium. A mental state of disequilibrium, in this case, would cause discomfort and uncertainty and, more importantly, instill an internal state of motivation, compelling a student to seek some form of resolution (Phan and Ngu, 2019). The cognitive growth of a student in a subject matter (e.g., the experience of mastery of Algebra of a secondary school student), according to Piaget (1963), entails his/her engagement in conflict resolution, which would transform a mental state of disequilibrium {e.g., the exposure of a student to an equation [e.g., *x*(*x* + 9)^2^ = −10] that she cannot solve} to a mental state of equilibrium (i.e., the student is now able to solve the equation).
iii. *Flow theory* of Csíkszentmihályi (1990, 1997) contends an internal desire for a person to experience a state of “flow.” Academically, for example, a 4th-year university student may enjoy and immerse in his thesis writing of the black holes (https://www.nasa.gov/vision/universe/starsgalaxies/black_hole_description.html) to the point where he misses his part-time work at local café. Cognitive flow, in this case, may reflect the state of intrinsic motivation of a student, his enjoyment and gratification and satisfaction, and, relatedly, his enriched experience of flourishing. Interestingly, however, we argue that successful experience of flow would require a perceived state of stability or, as Piaget (1963) details, a mental state of equilibrium. In other words, continuing failures of a student in a subject matter, which may closely associate with his/her perceived state of uncertainty, would undermine and/or negate the personal experience of flow.

In summary, as a preliminary step for analysis, we have considered three comparable reasons, which may offer an alternative perspective into the discussion of cognitive entrenchment (Dane, 2011; Schmid, 2017; Engelberg, 2018). Rather than being negative (e.g., cognitive entrenchment results in a state of inflexibility of a person to make changes), we argue that cognitive entrenchment may yield a number of noteworthy and positive outcomes for development (e.g., the motive of a person to develop a heightened state of self-efficacy). Situated fixation to a particular course of action may, in this sense, relate to some logical, well-rationalized reason that otherwise would not be possible—for example, could a student enjoy a state of cognitive flow if he was asked to “step outside” of his repertoire of knowledge, skills, and understanding?

## Advancing the “Positivity” of Cognitive Entrenchment

Let us advance the study of cognitive entrenchment (Dane, 2011; Schmid, 2017; Engelberg, 2018) by considering a conceptual analysis that may be positive and beneficial for students. Cognitive entrenchment, we contend, may account for the personal achievement of the following: (i) the seeking of a person of a perceived state of comfort, (ii) the desire of a person to achieve a state of optimal best efficiently, and (iii) the desire of a person to avoid a state of cognitive load imposition, which could weaken his/her performance outcome. We argue then that, unlike previous assertions, situated fixation to a course of action is encouraging and warranted and may serve a number of beneficial purposes, which we consider in this section.

### Comfort Zone and a Zone of Discomfort

The first line of reasoning for a need to cognitively fixate to a course of action relates to a theoretical concept known as a *comfort zone* (Brown, 2008; White, 2009; Liepold et al., 2013), or a *perceived sense of comfort*, which is defined as “a behavioral state within which a person operates in an anxiety-neutral condition, using a limited set of behaviors to deliver a steady level of performance, usually without a sense of risk” (White, 2009, p. 2). This definition contends that in the absence of a change in anxiety and/or any other negative emotion, the level of performance of a person would remain constant. In contrast, however, a change in the level of anxiety and/or any other negative emotion of a person would result in his/her performance—either upwards or downwards. For example, existing research has shown that anxiety is negatively associated with academic performance (Pajares and Kranzler, 1995; El-Anzi, 2005; Segool et al., 2013; Onyekuru and Ibegbunam, 2014). Positive emotions such as happiness, in contrast, are analogous to an improvement in academic performance (Spice, 2011; Tabbodi et al., 2015; Phan and Ngu, 2020).

A perceived sense of comfort (i.e., the comfort zone) is positive and may explain why individuals would choose to remain with the *status quo*—that is, individuals would fixate and capitalize on their expertise in order to achieve continuing success in a specified domain of functioning, giving rise to a perceived sense of comfort. In other words, a person's inner desire to maintain and sustain a level of comfort (i.e., his/her desire to be within a comfort zone) would compel and motivate him/her to not consider any form of deviation. Deviating from the *status quo* (e.g., the use of a pedagogical strategy of a student that she is knowledgeable and well-versed in), in this instance, may yield uncertainties and/or unknown results, which could instill a perceived sense of discomfort. For example, in the context of schooling, the state of apprehension, indecisiveness, and/or uncertainty of a secondary school student of potential discomfort could convince and compel her to remain with a known course of action and not to consider any change. Such consideration (e.g., the student chooses to use a particular cognitive strategy that she has mastered), in this analysis, would continue to bring success, resulting in positive emotions and feel-good experiences. In a similar vein, non-academically, the insistence of a football coach on remaining inflexible and entrenched with a training methodology may relate to his conviction and self-belief that such an act would yield continuing successes. Moreover, of course, there is also the viewpoint that inflexibility or unwillingness to change and to adapt to a new context is associated with a perceived sense of comfort—that a football coach, in this case, may feel content and more at ease (i.e., one characteristic of comfort) with his knowledgeable and well-versed training methodology.

It is interesting that in his recent writing, Brown (2008) referenced two contrasting “zones” that a person may experience, seek for, and/or avoid: a comfort zone vs. a panic zone. Natural tendency suggests that any person, for that matter, would seek a comfort zone (e.g., the experience of contentment and ease) and, where possible, to avoid a discomfort zone (e.g., the experience of anxiety and pessimism). We rationalize that a panic zone espouses negativity (e.g., instilling a perceived sense of apprehension), instability, and a state of uncertainty, limiting a student, say, from progressing forward in terms of his/her individual growth in a domain of functioning. A comfort zone, which is an antithesis of a panic zone, is desirable and may yield a number of positive outcomes. For example, in the context of schooling, a comfort zone, which espouses different types of positivity (e.g., instilling a perceived sense of feel-good experience and contentment), may provide a basis and/or opportunities for a student to flourish in a subject matter. In other words, “being” in a comfort zone is positive, favorable, and optimistic and would assist a student to experience, possibly, a state of personal contentment and equanimity, giving rise to his/her self-belief in personal resolve and conviction that changes are not required. We reason that a comfort zone in itself is motivational, providing grounding to encourage a student to maintain and sustain a course of action. Changing a course of action, in this case, could potentially result in the shifting of a student from a comfort zone to a discomfort zone.

### The Importance of Optimal Best

*Optimal best*, or optimal functioning, is a positive and motivational concept that closely aligns with a state of flourishing (Fraillon, 2004; Martin, 2011; Phan et al., 2016, 2020a). Seminal report of Fraillon (2004) on personal well-being introduced two interrelated concepts, known as “actual functioning,” denoted as L_1_, and “notional best functioning,” denoted as L_2_. This introduction of actual functioning and notional best functioning led to our comprehensive theoretical article (Phan et al., 2016), in which we expanded on initial ideas of Fraillon (2004) and conceptualized a theory termed as “levels of best practice.” We have undertaken a number of empirical studies (e.g., Phan et al., 2019b; Phan and Ngu, 2020, 2021b), which subsequently led to our revision and refinement of the 2016 theoretical-conceptual analysis article (Phan et al., 2016). Notably, for example, our recent conceptual analysis article (Phan et al., 2019a) provides an in-depth explanation of optimal best and, more importantly, our proposition of the *process of human optimization*. How does a person achieve L_2_, or notional best functioning in a subject matter?

**Figure 1:**
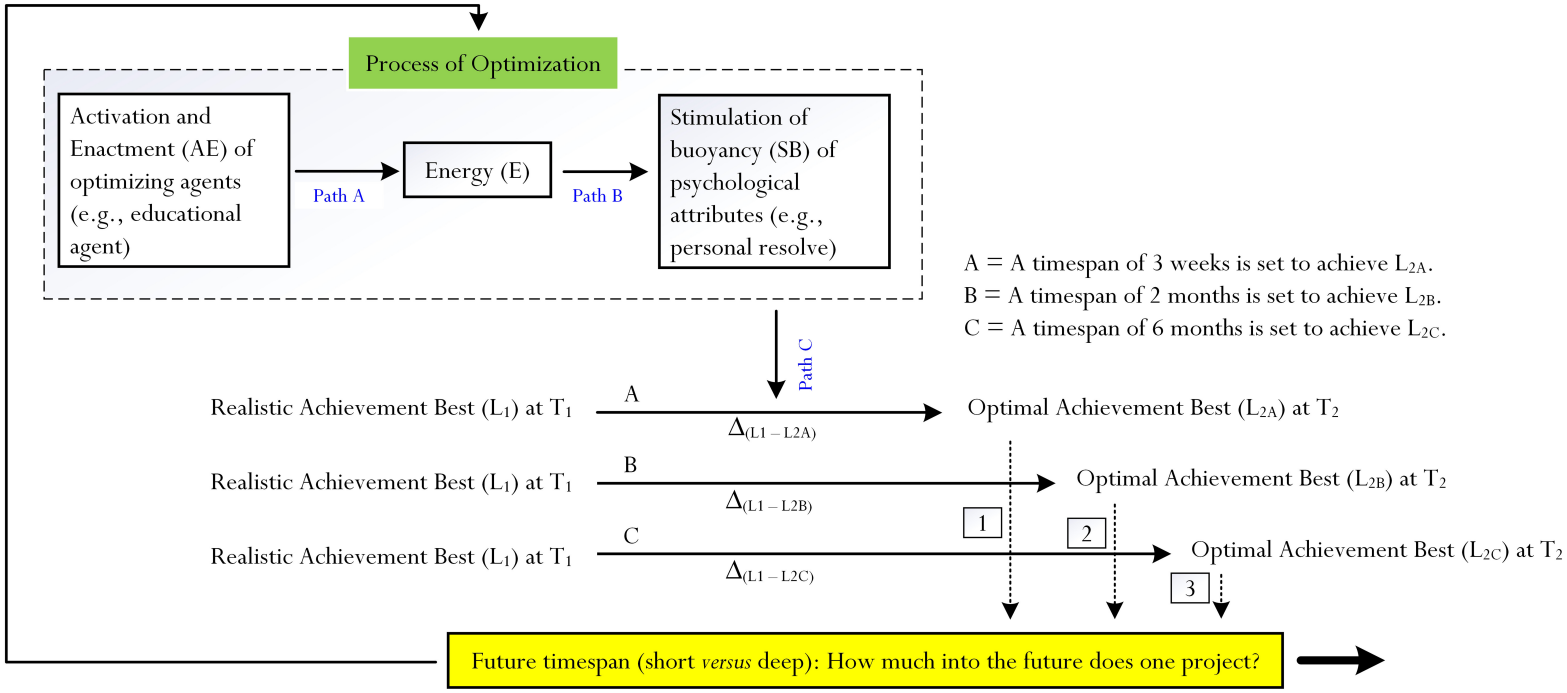
Process of optimization. Source: Phan et al. (2020a). From this figure, there are three examples of optimal best, which, in this case, differ in terms of perceived cognitive complexity: L_2A_ (e.g., perceived as being the easiest), L_2B_, and L_2C_ (e.g., perceived as being the most difficult). On this basis, there are three corresponding differences: Δ_(L1−L2A)_, Δ_(L1−L2B)_, and Δ_(L1−L2C)_.

Human optimization is a psychological process, which may explain and/or account for the achievement of a person of L_2_ from L_1_ (Phan et al., 2019a, 2020a). Our theoretical account of the psychological process of human optimization is summarized in Figure 1 and, in this case, depicts two major “levels of practice”:

i. *Realistic achievement best*, denoted as L_1_, and corresponds to actual functioning of Fraillon (2004), is defined as the current level of capability of a person (i.e., What can the person do at present?).
ii. *Optimal achievement best*, denoted as L_2_ and corresponds to notional best functioning of Fraillon (2004), is defined as the level of optimal capability of a person (i.e., What is the maximum in capability that the person can achieve?).

We theorize that L_1_ serves as a point of reference (e.g., a reference point of, say, “0”), which then may assist in the “calculation” and “quantitative derivative” of L_2_ (Phan et al., 2017, 2019a, 2020a). Evidence of a successful accomplishment and/or experience of a student of L_2_ (e.g., knowing how to solve simultaneous linear equations with two unknowns: 2*x* + *y* = 10 and *x* – *y* = 20), in part, entails a point of reference (i.e., L_1_), which a student could use and/or would use to strive for optimal best. For example, in the context of mathematics learning, consider the following:

- L_1_: the testament of a student that she can solve basic arithmetic problems—for example: 8 + — = −10 (Problem A).
- L_2_: the indication of a student that she can accurately solve simultaneous linear equations with two unknowns, *x*, and *y*—for example: 2*x* + *y* = 10 and *x* – *y* = 20 (Problem B).

In this case, the example indicates that the perceived cognitive complexity of Problem B exceeds that of Problem A (i.e., cognitive complexity of Problem B > cognitive complexity of Problem A). As such, the quantitative and/or qualitative difference between L_1_ and L_2_, denoted as Δ_(L1−L2)_, is positive and reflects the improvement, growth, and/or development in mathematics of a student. Moreover, from our recently published article (Phan et al., 2019a), successful accomplishment of L_2_ (i.e., the solving of Problem B), which equates to a positive difference between L_1_ and L_2_, +Δ_(L1−L2)_, reflects the experience of a student of a state of “flourishing.”

Successful accomplishment of L_2_ from L_1_, according to our theorization (e.g., Phan et al., 2017, 2019a, 2020a), requires some form of optimization. Referring to our recent comprehensive review (Phan et al., 2019a), the psychological process of optimization is intricate and consists of three major paths (Figure 1):

i. *Path A* (i.e., the activation of optimizing agents (e.g., psychological agent: self-efficacy, Bandura, 1997), which instills a state of energy, denoted as “E”).
ii. *Path B* (i.e., a state of energy, vitality, and liveliness) would initiate the stimulation of buoyancy of different psychological (e.g., effort expenditure, personal resolve) attributes).
iii. *Path C* (i.e., the buoyant experiences of different types of psychological (e.g., effort expenditure, personal resolve) attributes would, in turn, arouse and sustain the state of functioning of a person.

Optimization, reflecting the enactment of Path A, Path B, and Path C, may vary in intensity—that is, the magnitude of the strength of optimization, according to Phan et al. (2019a), may vary in accordance with the cognitive complexity of L_2_ or the difference between L_1_ and L_2_. Table 1 provides an example, which shows differences in perceived cognitive complexity. With reference to Figure 1, consider two secondary school students who have the same level of L_1_ knowledge and experience in linear equations (e.g., they can solve linear equations with one unknown, *x*). At present, the indication of Student A attests to his maximum capability to accomplish L_2A_, whereas the indication of Student B, in contrast, attests to her maximum capability to accomplish L_2B_. Consider another student, Student C, who also has the same level of L_1_ knowledge and experience but, in this case, reports on his maximum capability to accomplish L_2C_. In terms of perceived cognitive complexity, we note the following: L_2C_ > L_2B_ > L_2C_.

**Table 1:** L_1_, L_2_, and perceived cognitive complexity.

**Students**		Intensity of optimization^*^	**Perceived cognitive complexity**
	L_1_ *x* + 10 = −4	-	
Student A	L_2A_ 4(*x* + 5) = 3(*x* – 7)	0.25	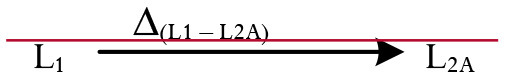
Student B	L_2B_ 4(*x* + 8)^2^ = 6	0.40	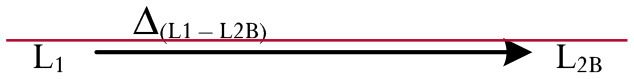
Student C	L_2C_ 4(*x* + 8)^2^ = 3(*x* – 7)	0.55	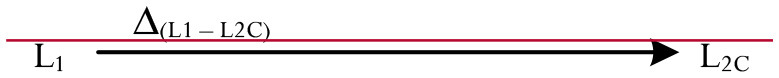

* *Arbitrary numerical value of the intensity of optimization, ranging from 0 (e.g., minimum optimization needed) to 1 (e.g., maximum optimization needed)*.

From Table 1 and Figure 1, and in tandem with the explanation of Phan et al. (2020b), we note that using L_1_ as a point of reference: the achievement of L_2A_ is the easiest, whereas the achievement of L_2C_ is the most difficult. Moreover, as shown in Table 1, the intensity of optimization is the highest for L_2C_ (Note: we have placed an arbitrary value of 0.55) and, in contrast, the lowest for L_2A_ (Note: we have placed an arbitrary value of 0.25). An interesting question then is how the successful accomplishment of optimal best relates to cognitive entrenchment (Dane, 2011; Schmid, 2017; Engelberg, 2018) and/or the concept of a comfort zone (Brown, 2008; White, 2009; Liepold et al., 2013)? We rationalize and posit that the successful accomplishment of L_2_, regardless of its perceived level of cognitive complexity, yields and reflects a state of comfort. In contrast, likewise, the inability and/or difficulty of a student in accomplishing L_2C_, say, would result in a state of discomfort. If this is the case, then we expect to find that many students, in general, would seek to achieve levels of optimal best (e.g., L_2A_ or L_2B_) that are easy and achievable. Achievable levels of optimal best, in this sense, are more likely to yield comfort and not discomfort. As such, the indication of a person of a level of optimal best for successful accomplishment would, in part, depend on his/her current level of best practice. For example, referring to Table 1, a relatively weak student (i.e., a low level of L_1_) would not indicate a complex level of optimal best (e.g., L_2C_). We posit that importantly:

- A knowledgeable and well-versed student (i.e., a high level of L_1_) would likely attest to his/her capability to successfully achieve a complex level of L_2_. A less knowledgeable student, in contrast, would indicate his/her likely success with a less complex level of L_2_.
- A knowledgeable and well-versed student is likely to capitalize on his/her level of L_1_ and not deviate from these personal experiences, as this sustaining of the *status quo* to achieve a state of L_2_ could potentially associate with a perceived sense of comfort.
- Excessive difference between L_1_ and L_2_ and, more importantly, one's quest to achieve a complex level of L_2_ with limited knowledge and understanding (i.e., a low to a modest level of L_1_) may, in this case, cause a state of uncertainty and, hence, a perceived sense of discomfort.
- A high level of L_1_ is beneficial and advantageous, serving as a point of reference for complex levels of best practice, which a student may accomplish. Moreover, a high level of L_1_ may instill confidence and self-belief of personal conviction that one can achieve a complex level of L_2_, resulting in his/her perceived sense of comfort.

## The Importance of Cognitive Load Imposition

As we theorize (Phan et al., 2017, 2019a), the achievement of optimal best requires the proactive enactment of the psychological process of optimization. One interesting premise for consideration, which we have detailed elsewhere, is the potentiality for *cognitive load theory* (Sweller et al., 2011; Sweller, 2012) to substantially account for the accomplishment of a person of L_2_. Cognitive load theory, in accord with the characteristics of human cognitive architecture, contends that effective cognitive processing of information is closely aligned with the concept of *cognitive load imposition* (Sweller et al., 2011; Sweller, 2012), which may assist in the appropriate design and development of an instructional approach for quality learning experiences. The main objective of an appropriate instructional design is to minimize the cognitive imposition of the working memory and take advantage of the prior knowledge of a person (if any) in the form of schemas in the long-term memory to facilitate skills acquisition. There are three “types” of cognitive load that could explain the importance of cognitive load imposition:

i. **Intrinsic cognitive load:** intrinsic cognitive load is imposed by the complexity of learning materials (i.e., how difficult or easy is the given task?). Differential levels of “element interactivity” act as contrasting indexes of cognitive complexity, whereas element interactivity accounts for the extent to which elements within a subject matter interact. Processing interactive elements and the relationship between them simultaneously in working memory to facilitate comprehension results in cognitive load imposition. Intrinsic cognitive load decreases with an increase in the expertise of a learner, and the opposite is also true (Kalyuga et al., 2003). Once a learner has gained expertise in a particular domain of functioning, the multiple interactive elements would then subsume to form a schema (i.e., a single element). For example, a mathematics teacher who possesses a schema for the equation of 2x + 5 = 12 would solve this with minimal, if any, conscious effort. On this basis, it is plausible to modify intrinsic cognitive load by changing either the level of element interactivity of the subject matter or the level of expertise (Sweller, 2010).
ii. **Extraneous cognitive load:** extraneous cognitive load is imposed by sub-optimal instructional designs that could, in effect, hinder learning and quality experiences. For example, worked examples that direct the attention of a learner to the problem state and the associated operators, in this case, imposes a lower level of the extraneous cognitive load than solving equivalent problems that involves a random search for solution options (e.g., Sweller and Cooper, 1985). It is important to focus on elimination, and the deterrence of extraneous cognitive load as this would, in effect, improve efficiency in the learning processes.
iii. **Germane cognitive load:** germane cognitive load is imposed by using working memory resources to deal with the intrinsic nature of the learning tasks. For example, in terms of academic learning, variability practice requires a student to distinguish a similar solution across different contexts (Paas and Van Merriënboer, 1994; Likourezos et al., 2019). Thus, unlike intrinsic and extraneous cognitive loads, the germane cognitive load does not exert an independent source of cognitive load; instead, germane cognitive load is regarded as part of intrinsic cognitive load. In other words, both intrinsic cognitive load and the extraneous cognitive load imposed on the working memory of a person during the course of his/her learning.

There is extensive research development, which has delved into the validity and applicability of cognitive load theory (Sweller et al., 2011; Sweller, 2012). For example, from an academic point of view, how does cognitive load theory assist educators to facilitate and/or encourage effective learning? There are different educational inquiries (Sweller et al., 1990; Sweller, 2010; Richland et al., 2017; Seufert, 2018), empirically and/or philosophically, which have been undertaken to explain the potency of cognitive load theory. One aspect of our research inquiries has focused on designing and organizing appropriate instructional designs for effective learning in mathematics (e.g., Ngu et al., 2015, 2016; Ngu and Phan, 2016b). In particular, from a cross-cultural perspective (e.g., Malaysian students vs. Australian students), we were interested in seeking clarity into the effectiveness of the *balance method* of learning vs. the *inverse method* of learning (Ngu and Phan, 2016a). One interesting rationale for us, in this case, relates to the potential complementary use of different instructional designs and/or pedagogical approaches to ensure a deep, clearer understanding of the subject matter.

**Figure 2:**
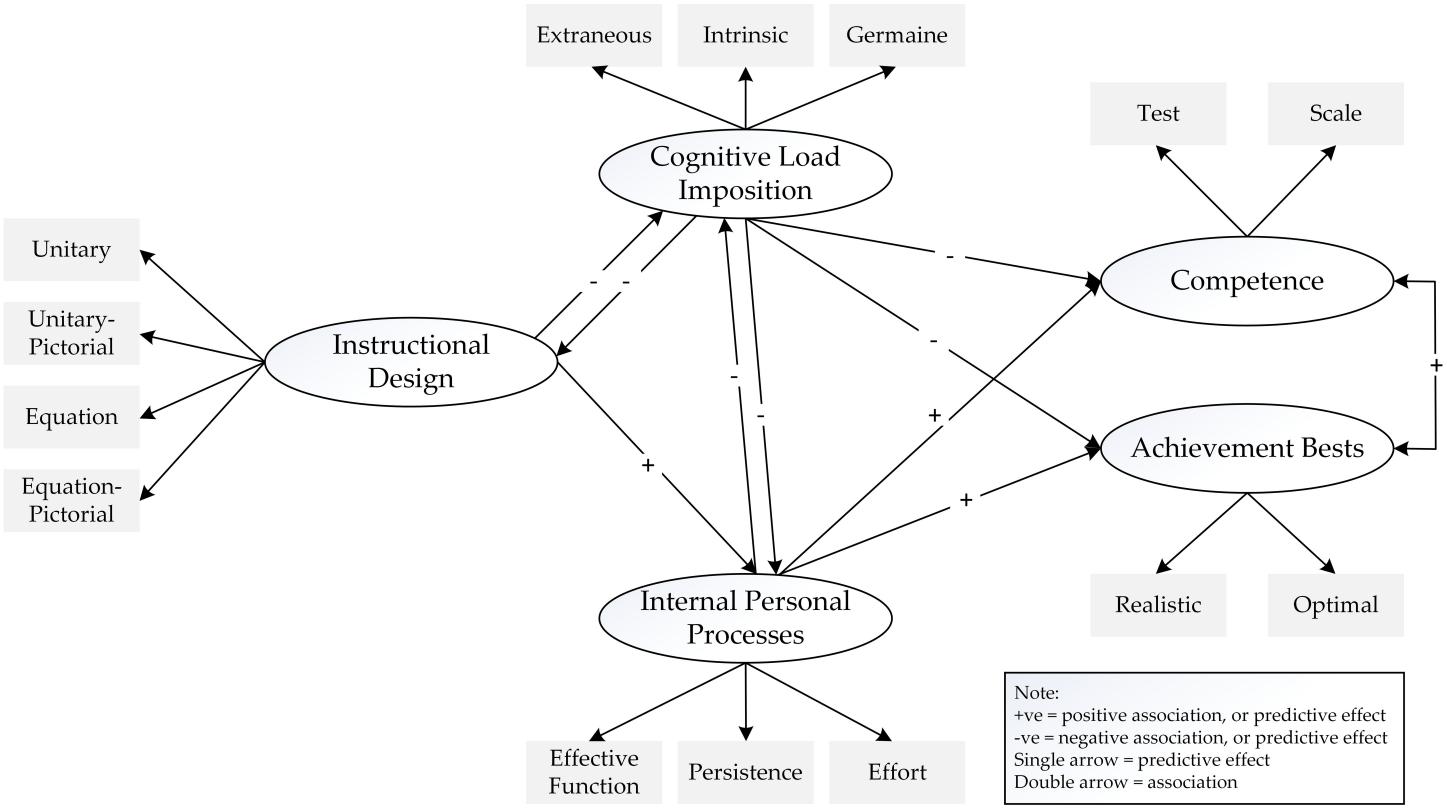
Interrelations between cognitive load, instructional designs, levels of best practice, and psychological processes. Source: Phan et al. (2017).

Over the past few years, we have made concerted attempts to explore and identify a potential association between cognitive load imposition and motivational beliefs. For example, does a high level of extraneous cognitive load weaken the internal state of a student of motivation for learning, say, mathematics? In line with our research development into the study of human optimization (Phan et al., 2019a, 2020a; Phan and Ngu, 2021b), we conceptualize and situate the notion of student motivation within the context of the theoretical concept of optimal best (Fraillon, 2004; Phan et al., 2016, 2017). Conceptually, in this analysis, we rationalize that the successful accomplishment of a person of optimal best in a specific domain of functioning (e.g., the mastery of deep, meaningful understanding of Buddhist meditation of a senior citizen) would reflect his/her state of intrinsic and/or extrinsic motivation (Phan and Ngu, 2021a). The inability of a person to achieve and/or to experience optimal best, in contrast, would indicate a low state of intrinsic and/or extrinsic motivation. More importantly, this consideration and how cognitive load imposition could associate with instructional designs and levels of best practice is shown in Figure 2.

The conceptualization depicted in Figure 2, which we published in *Educational Psychology Review* (Phan et al., 2017), is significant as it proposes a number of pathways for consideration (e.g., the negative impact of cognitive load imposition on an instructional design). Importantly, of course, the significance of this conceptualization lies in our attempt to unify three major theoretical orientations or frameworks within one structural model for examination: cognitive load imposition, optimal best, and instructional designs. For example, we propose that an appropriate instructional design (e.g., using the balance method) coinciding with a low level of extraneous cognitive load would help “optimize” the mathematics learning experience of a student. Such an enjoyable and enriching learning experience, in turn, would instill an internal state of personal resolve, conviction, and motivation for continuation. An inappropriate instructional design (e.g., the use of the inverse method), in contrast, would impose a high level of extraneous cognitive load, resulting in the sub-optimization of learning experiences (i.e., sub-optimal learning experience). In other words, our consideration entails a need to acknowledge and to recognize that cognitive load imposition (Sweller et al., 2011; Sweller, 2012) could feature centrally in a system of change, academically and/or non-academically.

Interestingly, in tandem with our inquiries into the nature of optimal best (Fraillon, 2004; Phan et al., 2016, 2017), we recently introduced another theoretical concept, which we believe could support the study of cognitive load imposition (Sweller et al., 2011; Sweller, 2012): *perceived optimal efficiency* (Phan and Ngu, 2021c). Perceived optimal efficiency, in brief, is denoted by the following:

$$\begin{array}{l} Optimal\;Efficiency\;\left(\text{OE}\right)=\frac{\text{Maximum Outcome }\left(\text{Max }-\text{ O}\right)}{\text{Minimum Expenditure }\left(\text{Min }-\text{ E}\right)} \end{array}$$

Perceived optimal efficiency, from the above, is defined as the ratio between the *maximum* outcome and *minimum* expenditure of time, effort, and/or resources, etc., of a person (e.g., convoluted cognitive thoughts about a subject matter without any form of resolution). In a similar vein, as an antithesis of perceived optimal efficiency, we also introduced a corresponding concept or term, known as *perceived inefficiency*, which is defined as being the ratio between the *minimum* outcome in a subject matter (e.g., sub-optimal performance in mathematics learning) and *maximum* expenditure of time, effort, and/or resources, etc. (i.e., the most amount of expenditure that would be needed). This ratio, or the definition of perceived inefficiency, is depicted as shown:

$$\begin{array}{l} Inefficiency\;\left(\text{IE}\right)=\frac{\text{Minimum Outcome }\left(\text{Min }-\text{ O}\right)}{\text{Maximum Expenditure }\left(\text{Max }-\text{ E}\right)} \end{array}$$

Achievement of optimal best is desirable but requires personal dedication and a serious investment of time and effort. Interestingly, in relation to our recent discussion (Phan et al., 2020a), we contend that successful or unsuccessful achievement of optimal best would intimately relate to a level of perceived cognitive complexity (i.e., cognitive complexity of L_2_). For example, optimal best that is relatively simple to accomplish would not take too much time, effort, and/or the use of resources, whereas, in contrast, complex optimal bests would require much more time, effort, etc. This consideration into the cognitive complexity of optimal best, we reason, may closely align with the concepts of optimal efficiency (Phan and Ngu, 2021c) and cognitive load imposition (Sweller et al., 2011; Sweller, 2012) and, more importantly, support our inquiries into the expert schemas of a person and his/her cognitive entrenchment. In this analysis, would spending ~100 h with an instructor achieve a state of L_2C_ for Algebra (i.e., Table 1), say, feel justified? This reflective question, which we introduced (Phan and Ngu, 2021c), emphasizes a person's analysis, assessment, and judgment of two interrelated entities: cost vs. outcome.

Does an outcome have *perceived values*, which would motivate and compel a person to strive for success? For example, in terms of academic learning of Social Sciences, a secondary school student may realize that achieving optimal best in Psychology is a noteworthy feat for her future career planning. On this basis, the student may decide to dedicate and prioritize her time, effort, etc., to satisfactorily satisfy and fulfill the objective of achieving optimal best in Psychology. In terms of perceived optimal efficiency, however, the question is whether the produced outcome would provide strong, logical justification for the cost involved? As we discussed, ambitious optimal bests, or optimal bests that have high levels of perceived cognitive complexity, would require dedication in time, effort, the use of resources, etc. To minimize the cost involved, for instance, one could attempt to reduce cognitive load imposition (e.g., a reduction of extraneous cognitive load) *via* different means—more organized subject contents, appropriate and more effective instructional designs, etc. In the context of the present article, the capitalization and use of existing schemas to assist with the minimization of the cost involved is a possibility. This mentioning, we contend, emphasizes one's inclination and/or determination to remain steadfast without any deviation from a course of action. Changing a course of action, which may reflect the intent of a student to show innovation and creativity, may give rise to personal experience of new knowledge, requiring additional expenditure of time and/or effort.

## Conceptualization: Cognitive Entrenchment, Optimal Efficiency, and Cognitive Load

Achievement of optimal best is a desirable feat but at what cost? This question of involved cost has daily relevance and applicability, emphasizing the self-awareness of expenditure of time and effort, resource utilization, excessive cognitive processing of information, etc., of a person. From this analysis, it is not simply a matter of seeking opportunities, pathways, means, etc. that could facilitate and/or motivate a person to strive for optimal best. It is important from our recent research development for individuals, institutions, organizations, etc., to consider cost *versus* outcome (Phan and Ngu, 2021c). Ideally, of course, we would like to achieve a state of O > E, which is efficient and more effective in terms of a person's usage of his/her time, effort, etc. In the context of academic learning, a student may capitalize on his/her prior schemas as “resources” to help minimize cost and, at the same time, to optimize new learning experiences. This capitalization of prior intellectual knowledge is advantageous, helping to minimize the need of a student to expend additional time and/or effort to master a subject matter. We speculate that limited knowledge, skills, and understanding would limit the progress of a person, giving rise to excessive scaffolding, academically or non-academically, and, hence, the potentiality for inefficiency.

Our interest relates to the nature of cognitive entrenchment (Dane, 2010, 2011) and the extent to which this psychological concept could serve to achieve a state of optimal efficiency. Cognitive entrenchment may produce a variety of benefits, especially when we consider the quest of a person to achieve a state of optimal efficiency. Unlike the theoretical premise of Dane (2010, 2011), in this case, we argue that the existing schemas of a person may negate a state of inefficiency and help him/her to achieve optimal learning experiences. In other words, fixating on a well-versed course of action (e.g., the insistence of a student on the use of a particular instructional design) without any consideration for change may reduce and/or minimize the need of a person to invest more time, effort, the use of resources, etc. in order to master new contents and/or understanding. Moreover, from our perspective, relying and/or fixating on a well-versed course of action is beneficial, potentially helping to reduce cognitive load imposition.

### Philosophical Positioning: Propositions for Consideration

**Figure 3:**
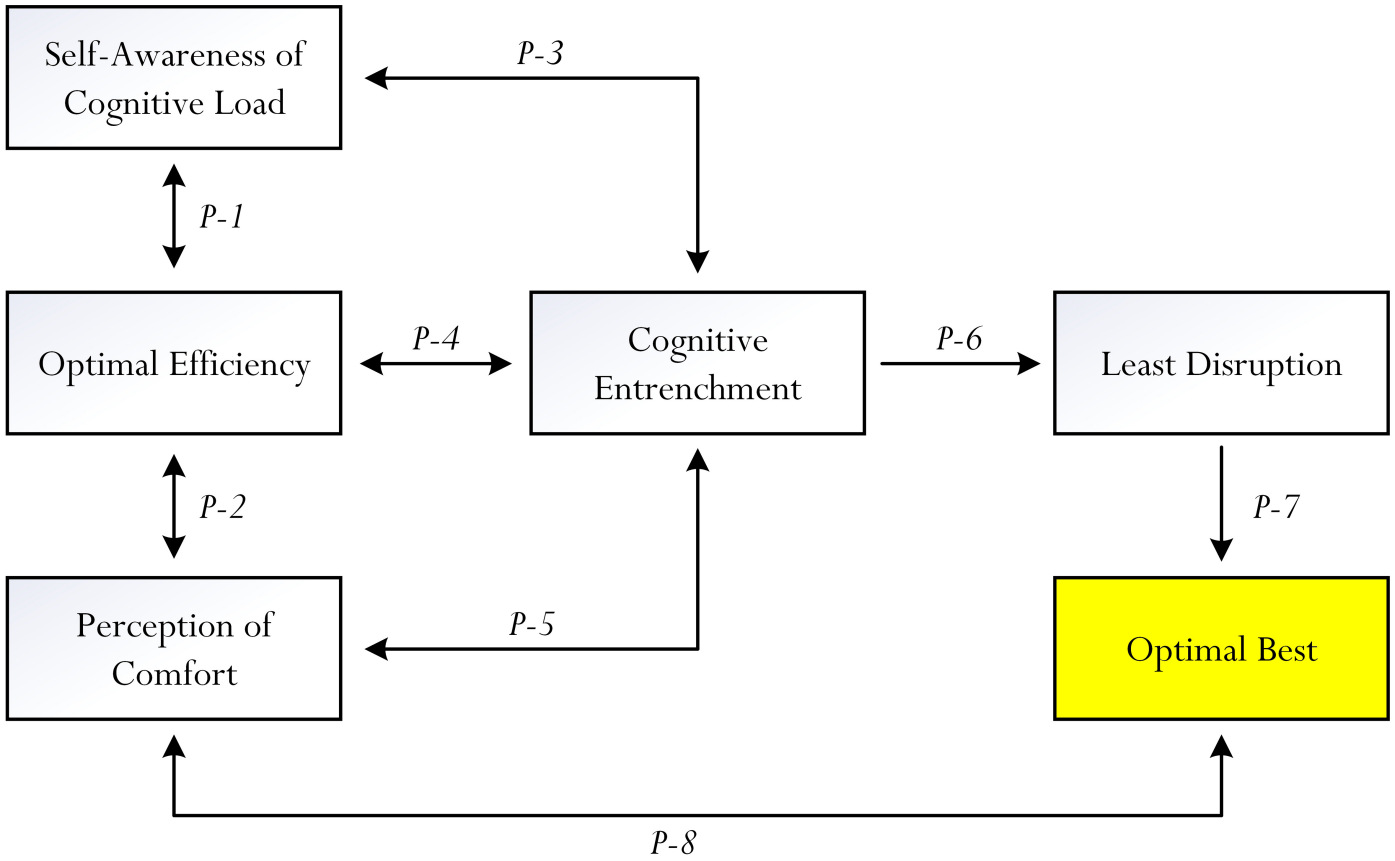
Conceptualized associations for consideration.

In this section of the article, we surmise our previous discussions and provide eight propositions (denoted as P1 – P8 in Figure 3), drawn from philosophical psychology and conceptualized reasoning, which may support the potent role of cognitive entrenchment. In this analysis, our propositions are different from those of Dane's (2010), offering an alternative insight into the importance and potential daily relevance of cognitive fixation. As shown in Figure 3, cognitive load imposition, a need for optimal efficiency, and the quest for experience of stability and comfort of a person, in tandem and/or individually, could account for the “positivity” of cognitive entrenchment.

In accordance with Figure 3, we postulate that the concept of cognitive entrenchment could act as a central variable, unifying self-awareness of cognitive load, optimal efficiency, and perception of comfort and exerting a positive effect on a theoretical concept, which we term as “least disruption.” Let turn our attention to the nature of the eight propositions and, more importantly, our rationalization as to why we firmly believe that there is the currency for the manifestation of cognitive entrenchment:

- **Proposition 1:** Proposition 1, denoted as P1, considers the association between self-awareness of cognitive load imposition and the achievement of optimal efficiency. This proposition contends the quest of a person to minimize his/her expenditure of time, effort, resources, etc., and to achieve optimal outcome in a subject matter (i.e., in other words, we want to achieve O > E). From the perspective of cognitive load imposition (Sweller et al., 2011; Sweller, 2012), an appropriate instruction that imposes low intrinsic cognitive load or extraneous cognitive load would lend support to the achievement of efficiency. In one of our recent studies (Ngu et al., 2016), we found that the equation approach of learning (i.e., the algebra approach) was more effective in helping secondary school students learn how to solve percentage change problems. Capitalizing on this study, let us consider a percentage change problem such as the following: “Last year, the car insurance premium was $500. It has increased by 5% this year. What is the new car insurance premium?” To solve this problem, one can formulate an equation such as:
  New car insurance premium = $500 + ($500 × 5%)
  By drawing on prior knowledge (i.e., existing schemas) of percentage quantity ($500 × 5%), a learner can process the percentage quantity as a schema, constituting a single element only. The processing of two elements [i.e., $500 and ($500 × 5%) in this case] would constitute low intrinsic cognitive load imposition, thus helping to facilitate optimal best for the percentage change problem with minimum effort. This example illustrates the importance of a novice having to capitalize on his/her existing schemas (i.e., percentage quantity), which then would assist in reducing the need for additional cognitive processing of new information (i.e., percentage change problem). Limited knowledge (e.g., the minimal understanding of percentage quantity of a person), in this case, would result in the student having to investigate and expend more effort, time, etc. Learners who have automated the specific schemas for percentage change problems, as we have noted (Ngu et al., 2016), are more able to solve transfer percentage change problems effectively. Let us consider an example: “Which is the better deal on an iPhone marked at $1,450?: (a) a discount of 10% or (b) a discount of 5% and then a further discount of 5%”. Solving this transfer percentage change problem would require the application of the percentage change schema twice. In line with cognitive load theory, once learners have automated the schema, the working memory can free up resources to adapt the solution procedure to solve transfer problems.
- **Proposition 2:** Proposition 2, denoted as P2, considers the association between perception of comfort and the achievement of efficiency. In line with the paradigm of positive psychology (Seligman, 1999; Seligman and Csíkszentmihályi, 2000; Csíkszentmihályi, 2014), a perceived zone of comfort is desirable as this envisaged “space” does not amount to any form of negativity (e.g., a state of anxiety). Natural tendency suggests that a person, in this case, would feel more inclined to seek a state of comfort and, likewise, to avoid a state of discomfort. For example, within the context of university studies, a 3rd-year undergraduate may choose to undertake Psychology courses simply because of his previous exceptional academic results. Exceptional academic results, in this case, may yield positive feelings (e.g., “feel-good” experience) and emotions (e.g., a state of happiness), giving rise to a perceived sense of comfort. Another academic subject may have contrasting negative experiences (e.g., repeated failures), resulting in a perceived sense of discomfort.
  We must consider the potential association between the perception of comfort (and, by contrast, discomfort) and the achievement of perceived optimal efficiency of a person. In contrast to that of inefficiency, evidence of optimal efficiency would espouse a perception of success (e.g., “I do need to spend so much time and yet I can achieve optimal…”), giving rise to the perceived sense of comfort of a person. However, evidence of inefficiency would convey messages of uncertainty, limited knowledge, and understanding, confusion and convoluted cognitive thoughts, etc., resulting in the perceived feeling of discomfort of a person. This proposition suggests an important awareness between two contrasting concepts—comfort vs. discomfort. The natural tendency, in this analysis, contends that the desires of individuals entail them wishing to seek and accomplish optimal efficiency, which would result from various enriched experiences of comfort. Inefficiency and a corresponding state of discomfort, unfortunately, required for some form of remedy and/or the use of preventive measures (e.g., the consideration of a person to transform a state of discomfort into a state of comfort).
- **Proposition 3:** Proposition 3, denoted as P3, considers the association between the self-awareness of a person of cognitive load imposition and his/her cognitive entrenchment in a subject matter. This proposition attempts to counter the argument of Dane (2010) and emphasizes the potentiality for cognitive entrenchment to minimize the imposition of cognitive load. For example, in relation to pedagogical practice, we note that the inverse method of learning multi-step linear equations is superior to that of the balance method, as the former imposes a lower level of cognitive load (Ngu et al., 2018). The main difference between the two pedagogical approaches lies in the understanding of a person of mathematical operations for solving linear equations (e.g., +3 on both sides versus – 3 becomes + 3). In this analysis, a student who has expert pedagogical knowledge and is well-versed, subject-wise, is more likely to choose an appropriate pedagogical approach purposively (e.g., using the inverse method of learning) that in effect would result in the minimization of cognitive load imposition.
  Novice knowledge is indeed detrimental and would require some form of “catch up” for students—that is, a student would need to invest some additional time, effort, resources, etc., to improve, advance, and/or acquire new theoretical understanding. As such, concerted attempts to seek new theoretical understanding in and of a subject matter would likely increase cognitive load imposition—for example, we reason that intrinsic cognitive load imposition would likely increase, consequently as a result of the limited understanding of a student of the subject matter and/or the complex nature of the subject matter at hand. In the absence of meaningful schemas (i.e., prior knowledge) for scaffolding, a student would have to invest more time, effort, and/or cognitive resources in order to help him/her master the subject content at hand. Aside from subject content knowledge, we speculate that limited pedagogical knowledge may also increase extraneous cognitive load, resulting in the perceived difficulty of a student in comprehending and understanding the use of a particular instructional design of a teacher.
  On this basis, situated fixation of expert schemas of subject content and/or pedagogical practice is advantageous, allowing a person to advance his/her learning with minimal difficulty. For example, learning to solve “algebraic transformation” problems poses a greater challenge for a student who has limited knowledge than learning to solve linear equations because the former would involve understanding the relationship between multiple variables. Advanced students, in contrast, would have the relevant and automated schemas in pedagogical practice, which then would enable and assist them to apply for a similar context (e.g., the well-versed understanding of a student of the inverse method of problem-solving in linear equations to algebraic transformation problems). As shown in Table 2, for example, the flexibility of the inverse operation allows a student to concurrently apply two inverse operations simultaneously, resulting in the ‘simplification and/or the generation of fewer solution steps (than the balance method). This example, we contend, illustrates the potentiality for the inverse method to impose a lower level of cognitive load, making it more efficient for effective learning than the balance method. On this basis, we firmly believe that the entrenchment of expert schemas may effectively serve to reduce and/or minimize cognitive load imposition.
- **Proposition 4:** Proposition 4, denoted as P4, considers the association between the achievement of optimal efficiency of a person and his/her cognitive entrenchment. Situated fixation in a subject matter and/or remaining on course without any deviation (e.g., a person's interest and/or consideration to try and adapt to a new situation) could result in and/or facilitate the continuing development of expertise of a person. On this basis, in-depth understanding of and in a subject matter is advantageous as this would allow a person to capitalize on this knowledge (e.g., existing schemas), which in turn could help to minimize and/or to reduce a need for additional resources, time, effort, etc. Limited knowledge, in contrast, would require a person to seriously invest in time, effort, etc., to seek personal understanding and master the subject content. Therefore, it is an encouragement in this analysis for a person to cognitively “fixate” and utilize his/her existing schemas to minimize inefficiency and to achieve a state of efficiency.
  It is, likewise, plausible to suggest that striving to achieve optimal efficiency could also motivate a person to acquire and advance in-depth knowledge in and of a subject matter. In other words, striving to achieve optimal efficiency may facilitate the self-awareness of a person that having in-depth knowledge is advantageous as this would help minimize and/or reduce a need for the continuation of expenditure of time, effort, etc. Novice learners, in particular, would benefit from the seeking of optimal efficiency as this personal focus could potentially instill a sense of purpose, personal resolve, and appreciation for the attainment of knowledge and understanding in and of a subject matter. Moreover, unconcern about inefficiency or achieving optimal efficiency could, interestingly, result in the feeling of indifference of a person in knowledge building and/or personal improvement.
- **Proposition 5:** Proposition 5, denoted as P5, considers the association between perception of comfort and cognitive entrenchment. There are a number of underlying reasons that could explain and/or support one's need to cognitively adhere to his/her existing schemas (Dane, 2010, 2011). One possible reason, as P3 suggests, relates to the self-awareness, recognition, and understanding of a person that cognitive load imposition is negative and detrimental. It is desirable and beneficial, as we detailed, to make a concerted effort to minimize cognitive load imposition. For example, in this case, a person's capitalization of his/her knowledge and understanding may help reduce the imposition of intrinsic cognitive load. Another reason, we contend, involves the desire of a person to seek a perceived state of comfort (Brown, 2008; Liepold et al., 2013), which is positive and may negate the personal feeling of angst, pessimism, and/or a perceived sense of helplessness. In the context of schooling, we propose that having expert schemas, or in-depth knowledge, would instill confidence and “feel-good experience,” strengthening the resolve and self-determination of a student to progress further without any sense of uncertainty and/or indecisiveness. Moreover, in this case, the availability of existing schemas is beneficial and advantageous, helping to instill a perception and feeling of comfort, contentment, ease, and security. This line of reasoning is analogous to the tenets of social cognitive theory (Bandura, 1986, 1997), which emphasize the potent role of a person's enactive learning experiences (e.g., past successes in a subject content) on the formation of his/her self-efficacy belief. Our argument contends that well-versed or expert schemas, forming the repertoire of the enactive learning experiences of a person, may similarly act as a relevant source of information in the formation of comfort.
  Personal feeling of comfort (e.g., the feeling of contentment), likewise, may serve as an important source of information, encouraging a student to remain on course and unchanged during his/her learning processes. A state of comfort, in this analysis, may serve to counter any personal need for change in terms of a student wishing to seek a new frontier, creativity, innovation, etc. Moreover, of course, a perceived state of comfort would closely associate with and/or facilitate the personal experience of flow (e.g., a state of cognitive flow; Csíkszentmihályi, 1997, 2014), strengthening the resolve and self-determination of a student to not deviate from a well-versed course of action. This interesting mentioning, delving into the positive nature of comfort may explain why some experts (e.g., the case of José Mourinho) choose to fixate on certain courses of action without any consideration for change. Perceived discomfort is negative and would, in this case, cause a state of uncertainty, demotivation, and pessimism, providing grounding for the case of cognitive entrenchment. Acquiring new knowledge, in this instance, would require a considerable amount of time, effort, the utilization of resources, and, more importantly, the possibility of personal experience of uncertainties, setbacks, and/or failures. If this the case, then we speculate that one would cognitively entrench rather than “dis-entrench” from an existing course of action.
- **Proposition 6:** Proposition 6, denoted as P6, considers the positive effect of cognitive entrenchment on a proposed theoretical concept, which we term as “the least amount of disruption” for a particular learning context. Maintaining and sustaining a course of action without any deviation, from our point of view, would result in and/or cause minimal disruption. A change in direction, whether short-term or long-term, in contrast, would likely cause disruption, which is negative and reflects and/or results in personal feelings of angst, chaos, uncertainties, obstacles, etc. More importantly, we content, disruption requires some form of resolution and stability, resulting in a person having to divert his/her focus of attention, time, effort, use of resources, etc. Interestingly, we refer back to our brief mentioning of the theory of personal constructivism of Piaget (1963, 1990), emphasizing the importance of cognitive stability. According to Piaget (1963, 1990), any state of cognitive conflict or “cognitive disequilibrium” would require some form of stability and personal resolution. In other words, when cognitive conflict arises (e.g., limited knowledge and inability of a student to find a solution for an Algebra problem), there is an instinct for a person to seek a logical and meaningful solution. On this basis, we argue that cognitive disequilibrium (Piaget, 1963, 1990) is deemed equivalent to a perceived state of disruption, requiring a person to engage in some form of resolution—that is, to consider some remedy or preventive measure that could, in effect, negate the personal experience of disruption.
  Reducing and/or minimizing disruption is encouraged as this positive feat would yield a number of benefits—for example, the perception, judgment, and feeling of stability Stability, the antithesis of instability, would facilitate and/or result in a perceived state of comfort. The opposite consideration is also plausible: that disruption in the environment gives rise to the feeling of instability of a person. Within the present article, we propose that cognitive entrenchment is advantageous and would help reduce and/or minimize the perceived sense of instability and disruption of a person. Creativity, innovation, and willingness to make changes could cause chaos and create a perceived sense of instability and discomfort. In contrast, of course, remaining on course without any consideration for change (e.g., the indication a student to use a particular instructional design) would bring stability and comfort, causing minimal disruption to a person. Adapting to a new context and/or a new course of action is not instantaneous and/or spontaneous and would, in this instance, require time, effort, and constant monitoring and evaluation. Moreover, any change and/or deviation could pose difficulties, especially disruption and imposition on a person's time, the focus of attention, etc. Even for experts, having to adjust and/or acquire new skills and knowledge for improvement and/or resolution purposes would amount to an increase in disruption, such as one's personal experience of cognitive load imposition (Sweller et al., 2011; Sweller, 2012).
- **Proposition 7:** Proposition 7, denoted as P7, considers the positive effect of a theoretical concept, which we term as “least amount of disruption” on the achievement of optimal best of a person. Minimizing disruption and maintaining stability, likewise, is positive and may produce a number of benefits and advantages. For example, referring to our earlier discussion, minimal disruption in academic learning may consist of minimizing a person's experience of cognitive load imposition when he/she updates existing schemas (Sweller et al., 2011; Sweller, 2012). Complex subject contents and/or a newly introduced pedagogical approach, in this sense, may impose cognitive load imposition, causing cognitive instability and classroom chaos in the learning process. Aside from cognitive load imposition, cognitive instability and classroom chaos may likely instill negative emotions (e.g., an increase in anxiety), which could weaken and negate one's quality learning experiences (e.g., the student, in this case, may purposively disengage from the learning process).
  In the context of classroom learning, unexpected mishaps cause chaos and likely disrupt the learning experiences of students, especially in terms of their motivational beliefs and aspirations to achieve optimal best practices. We contend that classroom disruption may serve to deter and/or to weaken one's personal experience of flow (Csíkszentmihályi, 1997, 2014), resulting in his/her inability to successfully achieve a state of optimal best. Negative extraneous influences (e.g., a teacher introduces a new topic for learning, which is unexpected) causing disruption require some form of remedy and/or rectification, which would amount to unnecessary expenditure of time, effort, resources, etc. A conducive state of calm, stability, and pleasantness, minimizing chaos and disruption (e.g., minimizing cognitive load imposition) is desirable and may play a prominent role in instilling confidence and motivation, guiding and facilitating students to strive for optimal learning experiences. Having said this, however, we ponder whether “purposive” disruption in class could give rise to some form of positivity. Purposive disruption (e.g., the purposive introduction of a teacher of a complex problem that students have not seen), we propose, may consist of a planned, deliberate “interjection” that is intended to motivate and/or to facilitate the aspiration of a person and to strive to achieve optimal learning experience.
- **Proposition 8:** Proposition 8, denoted as P8, considers the association between a person's perception of comfort and his/her achievement of optimal best in a subject matter. A zone of comfort (Brown, 2008; White, 2009; Liepold et al., 2013), from our point of view, is a positive entity, which may importantly reflect the internal state of contentment, satisfaction, easiness, etc., of a person. A perceived state of comfort likewise is likely to instill confidence and positive emotions (e.g., a state of situational happiness), guiding and motivating a person to progress, academically and/or non-academically, without any angst, uncertainty, pessimistic thoughts, etc. This consideration, importantly, we contend, supports our underlying premise, which posits the relevance and positive nature of the cognitive entrenchment. That cognitive entrenchment to a well-versed course of action, for example, may yield a state of comfort. A state of discomfort, in contrast, may align more closely with the feeling and experience of discontentment, dissatisfaction, uneasiness, etc., of a person. This personal experience of discomfort, we postulate, would cause angst and serve to negate a person's aspiration and/or ability to achieve optimal best in a subject matter.
  It is interesting to note that in his recent article, Brown (2008) adapted the theoretical model of Panicucci (2007) and outlined three different “types” of zone: a comfort zone, a growth/learning zone, and a panic zone. As the term connotes, a comfort zone contends a state of ease, contentment, and satisfaction, whereas, similarly, a growth/learning zone may espouse a state of willingness, motivation, and intellectual curiosity to seek new frontiers. A panic zone is similar to that of a state of discomfort and, differing from both comfort zone and growth/learning zone, espouses different forms of negativity (e.g., feeling highly anxious, consequently because he/she is not able to control and/or master the course of action at hand). Moreover, similar to that of a state of discomfort, a panic zone is counterproductive and would, in effect, weaken the quality learning experience of a person and, importantly, deter his/her achievement of optimal best. From this analysis, we speculate that a perceived positive zone in feelings and emotions would play a pivotal role in helping to situate a person to achieve his/her optimal best in a subject matter (Fraillon, 2004; Martin, 2011; Phan et al., 2016).
  Achievement of optimal best practice (Fraillon, 2004; Martin, 2011; Phan et al., 2016) is a positive feat and may, in this instance, provide testament of evidence of a person's pride, satisfaction, optimism, feel-good experiences, etc. Moreover, of course, pride, satisfaction, contentment, etc. are closely aligned with a perceived state of comfort, whereas sub-optimal experiences reflect a person's state of shame, dissatisfaction, pessimism, angst, etc., resulting in his/her perceived state of panic or discomfort. We speculate then that achievement of optimal best or, in contrast, sub-optimal learning experience would act as a potent source of information, conveying salient messages for a person to judge and assess—for example, in the context of schooling, achievement of optimal best in a subject matter and/or in a subject discipline would convey a message of comfort, which a student may choose to capitalize on further development. Similarly, the sub-optimal learning experience of a student in a subject matter may inform the teacher, the school, and/or relevant others that there is potential “evidence” of uncertainties, chaos, discomfort experiences, etc.

**Table 2:** Using the balance method and the inverse method to solve an algebraic transformation equation.

**Balance method**				**Inverse method**			
*p* – *x*	=	*c*	(– *p*) on both sides	*p* – *x*	=	*c*	(– *x* becomes + *x;* + *c* becomes – *c*)
– *p*		– *p*		*p* – *c*	=	*x*	
– *x*	=	*c* – *p*	(−1) on both sides				
(−1)		(−1)					
*X*	=	*p* – *c*					

## Summation

In summary, our descriptions of the eight propositions have been framed to support our justification for the position of cognitive entrenchment. Of course, we acknowledge that our construction of the eight propositions is exploratory and philosophical, relying on our use of theoretical psychology and personal reason-based reasoning. Ultimately, a question that we could ask is whether cognitive entrenchment is advantageous for development or whether it is detrimental and should be deterred? Can we soundly conclude that the inflexibility, inability, and/or unwillingness of a person to change contextually (i.e., cognitive entrenchment) is a negative feat? What are some underlying factors that could accurately account for the inflexibility, inability, and/or refusal of a person to change a course of action? In a similar vein, what are some logical reasons that could provide a counterargument, which may encourage and/or persuade a person remain steadfast and unchanged? Our discussion so far considers an alternative position for promoting and fostering cognitive entrenchment (Dane, 2010, 2011). There are three comparable aspects for consideration—namely:

i. Self-awareness of the potential negative impact of cognitive load imposition: A person's endeavor to minimize the impact of cognitive load imposition would weaken his/her quality learning experiences. In this analysis, remaining focused and capitalizing on existing schemas may potentially help to minimize the impact of intrinsic cognitive load. Flexibility and willingness to engage in creative and/or innovative changes could, in this instance, “misalign” with existing theoretical understanding, skills, and experiences, causing difficulties and problems for a person in terms of his/her progress. In the context of academic learning, for example, a student may capitalize on his/her existing understanding (i.e., his/her acquired schemas) and choose to focus on comparable subject content for studying (e.g., History when advancing from secondary school to university), as this similarity would help reduce intrinsic cognitive load. In a similar vein, referring to our previous football analogy, a football coach may prefer to use a familiar training methodology as this would cause less disruption in terms of his/her perceived cognitive load.
ii. The quest to seek optimal efficiency: It is desirable and more effective for a person to achieve optimal best practice, utilizing the least amount of time, effort, resources, etc. Unlike that of inefficiency, this quest to attain optimal efficiency is advantageous and beneficial as this would minimize the personal expenditure of human capital. We argue that fixation and remaining on course without any deviation for change in terms of innovation and/or creativity would, in this case, cause minimal disruption and, hence, assist a person to achieve optimal efficiency. The capitalization and entrenchment of existing knowledge, in this case, may help reduce and/or minimize instability and a person's cognitive conflict, resulting in a lesser need to invest in time, effort, etc. Attempts to deviate for change, creativity, and/or innovation, in contrast, could cause difficulties and problems (e.g., the difficulty of a student with comprehension and understanding of an Algebra problem, consequently as a result of her interest to explore an alternative pedagogical strategy), giving rise to a case of inefficiency (i.e., a student will have to invest in additional time, effort, etc. to confront and/or to master the change). On this basis, the cognitive entrenchment of existing knowledge may improve the minimization of time, effort, resources, etc.
iii. The importance of personal comfort: The positive perception and feeling of comfort of a person is paramount and may, in this case, associate with his/her perceived sense of stability (e.g., emotional stability) and low level of disruption. Personal comfort is a positive feeling, which may assist in motivating a person to aspire and strive for optimal best in a subject matter. A comfort zone is void of angst, pessimism, uncertainty, etc., providing grounding for flow and personal growth. Anything unstable and/or is perceived as being negative, in contrast, is more likely to associate with and/or yield different types of detrimental consequences—for example, in the context of academic learning, students withdraw and their sub-optimal achievement in a subject matter. There is credence to advocate for the concept of cognitive entrenchment, which we argue could account for the feeling of comfort of a person.

We argue that certain elements of cognitive entrenchment (e.g., the unwillingness to change of a person) are warranted and may assist a person to successfully adapt to his/her daily contexts. Overall, our eight propositions provide an alternative line of reasoning, detailing valid reasons and understanding as to why some individuals may choose to remain on course without any consideration for change. Moreover, however, we postulate that cognitive entrenchment in itself may associate with both positive and negative outcomes, depending on one's reason and position. We do not discount the fact that some may view the act of cognitive entrenchment itself (e.g., a person's unwillingness to change direction) as being “negative” (e.g., resulting in a person being not creative and/or innovative), limiting the advancement and individual growth of a person in a subject matter. However, we believe that capitalizing on one's existing knowledge and choosing to remain unchanged may help reduce cognitive load imposition and facilitate the achievement and personal experience of comfort and optimal efficiency.

## Conclusion and Future Directions

Why do some individuals think and act in a certain manner? Is inflexibility and/or an unwavering mindset an appropriate manifestation? Does a person's willingness to make changes to successfully adapt indicate his/her positive and proactive behavior? Does the exhibition of situated fixation make a meaningful contribution toward one's academic and/or non-academic progress? These questions, we contend, emphasize the salient nature of a person's situated mindset, which may yield a number of contrasting outcomes—for example: the inability to demonstrate creativity vs. the ability to achieve optimal efficiency. Importantly, as a point of reiteration, situated fixation to a course of action (e.g., the expert schemas of a person) may yield a number of advantages and benefits. In this sense, differing somewhat from conceptualization of Dane (2010, 2011), our rationale regarding cognitive entrenchment is positive and more favorable. Interestingly, this consideration offers an alternative viewpoint into the complexity of human agency (e.g., cognitive thoughts situated in context) and provides the theoretical grounding for further research development. We acknowledge that our discussions so far have been philosophical, drawing from personal conceptualization, and research-based rationalization (e.g., Dane, 2011; Schmid, 2017; Engelberg, 2018). As such, we are cognizant of the fact that the totality of our conceptualization lacks empirical evidence for support, limiting the merits of our rationale and argument for the positive case of cognitive entrenchment. For this section of the article, we want to discuss a few notable caveats and future directions noteworthy for development.

### Directions for Future Development

The study of cognitive entrenchment is of scholarly interest and has daily relevance for individuals, especially regarding their motivational beliefs, cognitive thoughts, structured and unstructured behaviors, etc. Our discussion so far has included a number of propositions, which may provide grounding for continuing research development into the positivity and negativity of cognitive entrenchment. Foremost, of course, is a recommended inquiry that could validate the extent to which a person's inclination to remain on course without any consideration of change (e.g., a student is fixated on his existing knowledge and skills) is associated, in this case, with his/her wish to: (i) maximize efficiency in the achievement of optimal best, (ii) minimize disruption and as such, to seek a state of comfort, and (iii) minimize cognitive load imposition, where appropriate. As Figure 3 reflects, this consideration requires the development of appropriate methodological and/or conceptual designs for implementation. In recent years, interestingly, a number of researchers have used *philosophical psychology* (Thagard, 2014; Phan et al., 2019a, 2020b) as a methodological paradigm to conceptualize new theoretical concepts, and/or to address clarification and to seek theoretical understanding into the complex nature of human agency. By all accounts, our described propositions are philosophical so far and require some robust form of methodology, which could help to scientifically validate the relevance and positivity of cognitive entrenchment.

In regard to the potential positivity of cognitive entrenchment in terms of the learning of complex tasks, Van and Jeroen (2013) and van Merriënboer and Kirschner (2018) have interestingly explored what is known as a “four-component instructional design model” (i.e., also known as the “4C/ID” model), which may assist in explaining the problem-solving process of complex tasks that target the development of expert performance. Specifically, the 4C/ID model considers problem-solving in real-life or authentic contexts that may inevitably involve *recurring* and *non-recurring aspects* of solution strategies. To encourage the development of the recurring aspects of problem-solving skills, learners are presented with just-in-time instructions and an emphasis on repetitive practice so that they could acquire automated schemas. To foster the acquisition of the non-recurring aspects of problem-solving skills, in contrast, learners are presented with supportive knowledge (e.g., reasoning, compare and contrast, decision-making) that helps explain the systematic approaches to solving the specific tasks at hand. Learners are required to learn to coordinate the recurrent and non-recurrent aspects of the problem-solving process to successfully learn how to solve complex tasks situated in real-life contexts. What is of significance then from this account is that our argument favors cognitive entrenchment, which focuses on a person having expert schemas for a specific category of problems. Research has indicated that the automation of expert schemas can facilitate the adaptation of the expert schemas to solve transfer problems in the same domain (e.g., Cooper and Sweller, 1987). Likewise, in regard to the 4C/ID model, it would be of interest to broaden the case for cognitive entrenchment to the context of the recurring aspects of problem-solving (i.e., procedural information) upon the acquisition of the non-recurring aspects of solving complex tasks. Nonetheless, for example, one possibility is that the 4C/ID model (Van and Jeroen, 2013; van Merriënboer and Kirschner, 2018) could “disprove” and “invalidate” our argument for the promotion of cognitive entrenchment for learning to solve the non-recurring aspects of complex tasks.

Another interesting line of inquiry that is of significance for advancement is concerned with the design and development of complementary conceptualizations that could substantiate our aforementioned propositions. For example, using philosophical reasoning and/or theoretical psychology could offer logical insights and help to elucidate the significant nature of cognitive entrenchment (e.g., the negative connotation of cognitive entrenchments, such as a person's unwillingness to adapt to a new situation). This recommendation emphasizes a researcher's logic, understanding, and reasoning to provide a sound conceptualization of a mapping of interrelations between cognitive entrenchment, optimal efficiency, cognitive load imposition, etc. In this analysis, aside from scientific evidence, additional psychological and/or philosophically derived conceptualizations could add credence and substantiate our postulation into the “positivity” of cognitive entrenchment. We recently advance our research inquiries into the topic of cognitive entrenchment (Dane, 2010, 2011) by considering an alternative concept, which we term as the “zone of cognitive certainty” (i.e., the envisaged zone of positivity a person) and the “zone of cognitive uncertainty” (i.e., the envisaged zone of negativity a person). Specifically, advancing the study of cognitive entrenchment, we speculate and argue that the *perception of certainty* of a person (e.g., that he/she is likely to be able to successfully adapt to a new situation) or *uncertainty* (e.g., that he/she is unlikely to be able to successfully adapt to a new situation) could govern and account for his/her cognitive thinking, action, and behavior.

In terms of empirical evidence, it would be of interest for researchers to consider incorporating reflections of individuals of their personal experiences, knowledge, skills, and/or understanding as “proxy” evidence, which could complement and/or substantiate the use of philosophical reasoning and theoretical psychology for research development purposes. In this instance, we rationalize that the position of a researcher, especially in terms of his/her assessment, could play a significant role in helping to warrant and justify his/her conceptualization. For example, drawn from our propositions (i.e., P1 – P8) described earlier, we rationalize two corresponding and contrasting viewpoints: (i) perception of certainty (e.g., that he/she is likely to be able to successfully adapt to a new situation) is closely aligned with the perceived positivity of cognitive entrenchment vs. (ii) perception of uncertainty (e.g., that he/she is unlikely to be able to successfully adapt to a new situation) is closely aligned with the perceived negativity of cognitive entrenchment. Moreover, reflecting our recent theorization of *holistic psychology* (Phan et al., 2021), we postulate that perceived positivity of cognitive entrenchment and perceived negativity of cognitive entrenchment would co-exist on opposite ends of a continual spectrum. In essence, our proxy evidence at this stage is preliminary and, by all account, from our recommendation, requires contributions from contrasting assessments, judgments, decision-making, etc., of other researchers.

## Author Contributions

Both authors contributed equally to the articulation and write-up of this manuscript.

## Conflict of Interest

The authors declare that the research was conducted in the absence of any commercial or financial relationships that could be construed as a potential conflict of interest.

## Publisher's Note

All claims expressed in this article are solely those of the authors and do not necessarily represent those of their affiliated organizations, or those of the publisher, the editors and the reviewers. Any product that may be evaluated in this article, or claim that may be made by its manufacturer, is not guaranteed or endorsed by the publisher.
